# Exploring the utility of robots as distractors during a delay-of-gratification task in preschool children

**DOI:** 10.3389/frobt.2023.1001119

**Published:** 2023-04-05

**Authors:** Jaishankar Bharatharaj, Irene M. Pepperberg, Senthil Kumar Sasthan Kutty, Achudhan Munisamy, Chris Krägeloh

**Affiliations:** ^1^ PAIR Lab, Bharath Institute of Higher Education and Research, Chennai, India; ^2^ Department of Psychological and Brain Sciences, Boston University, Boston, MA, United States; ^3^ PAIR Lab, Department of Psychology and Neuroscience, Auckland University of Technology, Auckland, New Zealand

**Keywords:** preschoolers, delay of gratification, self-control, executive function, robots, single-case experimental design

## Abstract

The development of executive function (EF) in children, particularly with respect to self-regulation skills, has been linked to long-term benefits in terms of social and health outcomes. One such skill is the ability to deal with frustrations when waiting for a delayed, preferred reward. Although robots have increasingly been utilized in educational situations that involve teaching psychosocial skills to children, including various aspects related to self-control, the utility of robots in increasing the likelihood of self-imposed delay of gratification remains to be explored. Using a single-case experimental design, the present study exposed 24 preschoolers to three experimental conditions where a choice was provided between an immediately available reward and a delayed but larger reward. The likelihood of waiting increased over sessions when children were simply asked to wait, but waiting times did not increase further during a condition where teachers offered activities as a distraction. However, when children were exposed to robots and given the opportunity to interact with them, waiting times for the majority of children increased with medium to large effect sizes. Given the positive implications of strong executive function, how it might be increased in children in which it is lacking, limited, or in the process of developing, is of considerable import. This study highlights the effectiveness of robots as a distractor during waiting times and outlines a potential new application of robots in educational contexts.

## Introduction

Among self-regulation skills, delay of gratification (DG) is considered as one crucial to be fostered during child development (Royle, 2010) and interventions that broadly train children to restrain impulsivity seem to have the greatest effect on fostering improved executive function (EF, e.g., see Zelazo et al., 2018). Here we propose a novel means to enhance DG, based on the introduction of robots. We begin with a very brief review of research on DG, emphasizing previous technological interventions.

Mischel et al. (1989) referred to the ability to postpone “immediately available gratification in order to attain delayed but more valued outcomes” (.933) as future-oriented self-control or goal-directed DG. The work by Mischel and his colleagues has been highly influential in psychology and education, resulting in many experiments that explored the circumstances under which children are more likely to exhibit self-control. In a typical DG experiment, children are provided with a choice between 1) An immediately available, less preferred reward and 2) A more preferred, but more delayed one. Mischel and Ebbesen (1970), for example, presented preschool children with cookies and pretzel sticks and asked what they preferred. After each child had expressed their preference, the experimenter announced that they will leave the room for an unspecified amount of time. They also explained that, if the child eats the less preferred item during the time period, the experimenter will return immediately and the preferred item will no longer be available. If, however, the child waits until the experimenter returns on their own accord, the child will be able to eat the preferred item (or, in a variant, a larger amount). Because rewards often involved one *versus* two marshmallows (e.g., Mischel et al., 1972), this experimental procedure was later popularly referred to as the “Marshmallow Test” (Goldberg, 2008).

An important characteristic of the DG procedure is that the commitment to wait for the delayed reward is determined by the participant. As Shoda et al. (1990) described it, the delay and thus the frustration of the waiting period is self-imposed. It is not uncommon to observe participant-initiated distraction strategies to help resist the temptation to interact with the non-preferred item during the waiting period (Neubauer et al., 2012). The effects of teaching distraction strategies have also been explored in experimental studies. When Mischel et al. (1972) instructed children to think of something fun while waiting, the mean waiting time of the participants clearly increased. In an external distraction condition, children had a toy available to play with during the waiting time, which also substantially increased the likelihood of DG.

Much of the popularity of the DG paradigm is a consequence of longitudinal studies that reported associations between performance in self-control tasks during childhood and positive outcomes later in life. For example, Eigsti et al. (2006) linked efficient attention regulation in preschoolers during a DG task with enhanced cognitive control 10 years later; other longitudinal outcomes of enhanced self-control at preschool include lower body mass index 30 years later (Schlam et al., 2013). Research studies have found relatively stable and long-lasting associations between DG task performance as a child and behavioral measures as well as frontal-lobe functioning up to 40 years later (Casey et al., 2011). Length of waiting times in preschoolers also predicts academic competencies in adolescence (Shoda et al., 1990), and children with high waiting times were later described as more intelligent and attentive (Funder et al., 1983). Similar findings have also been observed in other cultural contexts (Li, 2022) as well as in cross-sectional studies using self-report measures of self-control (Tangney et al., 2004). Given the importance of such self-regulation in childhood for future success, educators have been increasingly interested in innovative methodologies that might strengthen these skills.

Notably, recent technological advances have had a profound influence on the manner in which psychosocial interventions are being provided to children in many areas but not specifically in self-regulation. For example, the ubiquitous nature of tablets and smartphones have made apps a cost-effective approach to deliver teaching material and to aid in the provision of social support (Krägeloh et al., 2016; Ismaili and Ibrahimi, 2017; Yousaf et al., 2020). However, this technology is very limited in its ability to offer an interactive experience for the user. A progression in technological functionality is therefore the use of robotics, which is aimed at creating an agent with whom the user can interact. Some robotic applications provide companionship, such as the seal-like robot *Paro* that has been linked to increased psychological wellbeing in older adults (Wada et al., 2009; Robinson et al., 2013). Other purposes of robotic technology are educational, such as for teaching academic and psychosocial skills to children with autism (Bharatharaj et al., 2017a; b), inclusive play in visually impaired children (Metatla et al., 2020), or computational thinking and improving STEM attitudes (Zhang et al., 2021). Nevertheless, the one area that has so far received relatively little attention is research on robotic applications in the teaching of DG.

Although several studies in the area have mentioned the terms “self-control” or “delay of gratification” (DG), these projects have implemented procedures that are very dissimilar to the classic Marshmallow Test. For example, Holtz and Lehman (1995) introduced young children to a clown box that functioned a bit like a robot. In this resistance-to-distraction study, the children were instructed to work on a task and ignore the temptation to interact with the clown box. Other studies (Carlson, 2005; Lillard et al., 2015) have used robots in a Forbidden Toy task (Lewis et al., 1989) that investigated to what extent children can resist the temptation to play with a robotic toy in the absence of the experimenter when explicitly having been instructed not to do so. Ferrari et al. (2009) described a methodology of teaching self-regulation skills to children with autism. Children first learn to press buttons on a robot for sensory rewards such as light flashes and robot movement. Then, in a multiple-player collaborative game, children are allocated a specific button that only they can press for a sensory reward. However, in order to obtain the robot’s reward in this variant of the game, children are required to press their button in turn with another child, thus teaching them to learn self-regulation skills. Holeva et al. (2021) investigated the effects of robot-assisted relaxation training for children and its effects on emotional self-regulation. Lastly, Silva et al. (2018) used a robotic dog as a control for novelty effects as children with autism interacted with a live dog. After exposure to the live or robotic dog (depending on the experimental condition), children were asked to resist the temptation to grab a preferred food item placed within their reach for 3 min, and some behavioral and physiological measures were taken during this period.

Although the procedure of Silva et al. (2018) is closest to the Mischel paradigm, the experimental task did not involve a choice between a delayed preferred option and an immediately available, less preferred one. Research thus far has not explored the utility of a robotic intervention or exposure to robots within the context of self-imposed DG. Because of its emphasis on the importance of learning to deal with frustration during the waiting period and learning to resist the temptation to yield to the impulsive option, the Marshmallow Test provides a fruitful approach to explore the potential for robotic interventions as young children develop self-regulation skills. Given the effectiveness of distraction to extend waiting times (Mischel et al., 1972; Putnam et al., 2002), the present study explored to what extent exposure to robots may function as an effective distractor from the temptation to choose the immediate, smaller reward. In repeated sessions, 24 preschool children were given a choice whether to immediately ingest one serving of a preferred snack item or to wait 5 min for a double serving. Depending on the experimental condition, 1) No teaching was provided, nor was play suggested during the waiting period, 2) Teachers encouraged playing with toys or sang songs with children, or 3) Children were able to observe and interact with several types of robots. Latency to choose the smaller amount of snack was used as a measure of DG and compared across conditions. Using a single-case experimental procedure (Barlow et al., 2009; Tate and Perdices, 2019), we tested the within-participant effect of the interventions in each of 24 participants. We hypothesized that the interaction with robots would increase waiting times and thus further increase DG compared to the other conditions. The purpose of the present study, however, was to investigate the feasibility of a robotic intervention within a multidisciplinary context of robotics, education, and psychology.

## Methods

### Participants

The experiment took place at a preschool near the university of the first author. Participants were recruited using cluster sampling to achieve an approximately even distribution by sex and age and to ensure that participation was spread around the various preschool classes, enabling the study to be conducted within the space and time constraints outlined by the preschool teachers. Inclusion criteria required participants to be able to understand and respond to instructions in English and not to have any diagnosis for an intellectual disability. Among the 29 participants initially recruited, data from 5 children were eventually excluded due to non-participation in more than one session in any of the three experimental conditions or due to parents’ request to exclude students from the study. Of the 24 participants, 11 were female and 13 male (Table 1). None of the participants had previous exposure to robots, except for P3, who had previously interacted with a Lego robot.

**TABLE 1:** Age and sex of the participants.

Participant ID	Age in years	Sex
S1	5	Male
S2	5	Male
S3	5	Female
S4	5	Female
S5	5	Male
S6	5	Female
S7	5	Male
S8	5	Female
S9	5	Female
S10	4	Female
S11	5	Male
S12	5	Male
S13	4	Male
S14	4	Male
S15	5	Female
S16	5	Female
S17	5	Male
S18	5	Female
S19	5	Female
S20	5	Female
S21	5	Male
S22	4	Male
S23	5	Male
S24	5	Male

### Procedure

The research was advertised on school notice boards where the study was conducted. A session with parents was organised by the school management to introduce the research objectives. An information sheet detailing the objectives of the study was also given to the parents, who all gave signed consent for their child’s participation to the school principal. No financial remuneration was provided. Prior to commencing the study, information was obtained about each child’s preferred sweet or snack.

The participants were grouped into three teams using simple random sampling. All sessions were conducted between 10 and 11a.m. at their classroom. The participants were seated at a table. Each participant’s favorite snack was placed in a bowl in front of them and they were told that they could wait for 5 min to get double the reward amount or choose to consume the smaller reward without waiting. Three teachers working at the school used a stopwatch to time the latency between the start of each session and when the children indicated, by tapping the table, that they would like to consume a reward. Three research assistants assisted the teachers with the procedure, recording the results on notepads.

The study was conducted over a period of 3 weeks with three sessions a week. Each block of three sessions was part of a different experimental condition. In Condition 1, no intervention was provided. Here, the participants were simply informed that they would need to wait for 5 min for the larger reward. Alternatively, they could choose to tap the table at any time and collect the reward available in front of them. In the first session of this condition, the teachers left the room and monitored the activity remotely. In the second session, one teacher was present in the room but had no interaction with children. For the final session of Condition 1, two teachers were present in the room and again had no interaction with the children. Participants who waited for 5 min were rewarded double the amount of snack and also received applause from other participants. In Condition 2, the teachers helped children to potentially increase their wait time by telling stories (Session 1), enabling access to the children’s preferred toys (Session 2), and saying nursery rhymes (Session 3). In Condition 3, children were exposed to a humanoid robot, Nao (Shamsuddin et al., 2011, for a review), and a commercially available tele-presence robot with a display screen (Reis et al., 2018). This tele-presence robot was controlled using an android application to navigate the robot and play videos on the screen. In the first session of that condition, the robots were placed in the room, completely inactive, and the children were not permitted to have physical interaction with them. In the second session, the robot performed actions including locomotion, singing, and playing rhymes on the screen. However, as in Session 1, the children were not permitted to physically interact with the robots. In the third session, the Nao robot was programmed to move around and speak a few words including “Hi kids, my name is Nao, and I am a humanoid robot” and “I am here to help you get more reward” and performed dance movements. The telepresence robot was navigated using the application, and videos were played on the screen. The children could interact with the robots during the performance such as dancing with Nao and asking the telepresence robot to play their favourite rhymes. This study was approved by the first author’s institutional ethics review board.

### Data analyses

Given the comparatively large sample size for a single-case experimental design, results were presented in a tabular form as opposed to individual graphs. Of interest were the changes in latency scores from Condition 1 to 2 and from Condition 2 to 3. To interpret effect sizes, a number of measures are available for single-case research (Parker et al., 2014; Rakap, 2015). As the measures *non-overlap of all pairs* (NAP) and TAU-U are considered superior among those (Manolov et al., 2016), they were selected as the effect-size measures for the present analyses. These non-parametric measures do not require any assumptions regarding the distribution of data and are considered to provide strong statistical precision (Parker et al., 2014).

To calculate NAP for each participant, each data point in the first condition was compared to each data point of the subsequent condition, thus yielding a total of nine comparisons (as there were three sessions in each condition). The number of times that a score in the second condition was higher than that of a data point in the first condition was calculated (i.e., number of increases in latency). A tied score was counted as 0.50. NAP was then calculated as the sum of A) the instances that the latency increased and 2) the scores for ties, divided by the total number of comparisons. For example, if there were six instances where a score in the second condition was higher than that in the first condition, and if there was one tie, NAP was 6.50/9.00. In cases of missing data, the measure was still calculated but only with the maximum number of comparisons that was possible. All calculations were conducted using Microsoft Excel.

TAU-U is an extension of the measure TAU_novlap_. The latter is calculated in a similar way as NAP. However, instead of adding tied scores, TAU_novlap_ is calculated by subtracting instances of score decreases from the increases, yielding a score *S*. This *S* score is then divided by the total number of comparisons (i.e., 9). For example, if a latency score increased five times but decreased four times, *S* was 1, and TAU_novlap_ was thus 1/9. TAU-U has the advantage that trends in the baseline phase (i.e., the first condition in any comparison analysis) are accounted for. The *S* score is thus adjusted by subtracting from it a *S* score obtained from separate trend analysis of the first phase. TAU-U does not require this trend to be linear (Parker et al., 2011).

For NAP, Parker and Vannest (2009) offered the following cut-off ranges: 0.00 to 0.65 for weak effects, 0.66 to 0.92 for medium effects, and 0.93 to 1.00 for large effects. The same cut-off scores have been proposed for TAU-U, where less than 0.65 has been described as an indication that the intervention was ineffective or questionable, a range of 0.66–0.92 indicating that it was effective, and above 0.92 very effective (Rakap, 2015).

## Results

The individual participant latency results by session and experimental conditions are shown in Table 2. Because the maximum waiting period was 5 min, latencies could vary from 1 s (immediate consumption of the snack) to 300 s (waiting until the end of the 5-min period). In Condition 1 (no active intervention), 3 of the 24 participants reached the maximum latency–one during the second session and two during the third session. In Condition 2 (standard teaching), four participants presented with a latency of 300 s, and six participants did so in Condition 3 (robot intervention). The proportion of missing data (from children who were absent from a particular session) was highest in Condition 1 and lowest in Condition 2. For Condition 1, latency scores significantly increased from Sessions 1 to 3 (repeated measures ANOVA, *F*(2) = 5.56, *p* < .01) but not for Condition 2 (*F*(2) = 0.14, *p* > .05). For Condition 3, scores again increased significantly across sessions (*F*(2) = 10.60, *p* < .01).

**TABLE 2:** Response latency (in s) for each participant by session and experimental condition.

	Condition 1 (no intervention)			Condition 2 (standard)			Condition 3 (robots)		
	Session 1	Session 2	Session 3	Session 1	Session 2	Session 3	Session 1	Session 2	Session 3
S1	5	2	1	1	1	1	32	58	44
S2	104		135	124	105	177	209	131	231
S3	135	198	192	229	205	156	270	240	
S4	92	119		175	120	68	229	201	
S5	265	216		171	209	165	171	189	222
S6		1	1	241	216	242	217	212	269
S7	108	136	39	276	299	264	240	253	285
S8		152	187	1	1		212	227	191
S9	1		1	227	187	216	49		1
S10	237		216	54	91	7	230	241	
S11	98	166	300	300	65	104	247	269	300
S12	9	300	241	35	300	47	125	269	274
S13	8	77	127	11	5	53	97	95	251
S14	97	217	300	25	128	300	215	300	300
S15	254	215	281	198	254	168	259	215	285
S16	248	239	278	215	276	300	45	158	218
S17	123	181	272	248	254	247	300	254	300
S18	8	5	3	1	5	9	32	7	45
S19	278	163	195	185	201	132	292	300	287
S20	29	27	116	13	32	117	65	187	139
S21	46	69	89	300	245	41	5	195	215
S22	27	40	64	145	3	38	284	300	300
S23	84	62	135	71	11	49	21	300	300
S24	8	19	26	43	16	146	97	165	293
Mean	102.91	124.00	145.41	137.04	134.54	132.48	164.29	207.22	226.19
SD	95.38	90.36	106.89	105.68	109.09	95.80	99.75	78.32	92.97
Median	94.50	136.00	135.00	158.00	124.00	132.00	210.50	215.00	269.00

**FIGURE 1:**
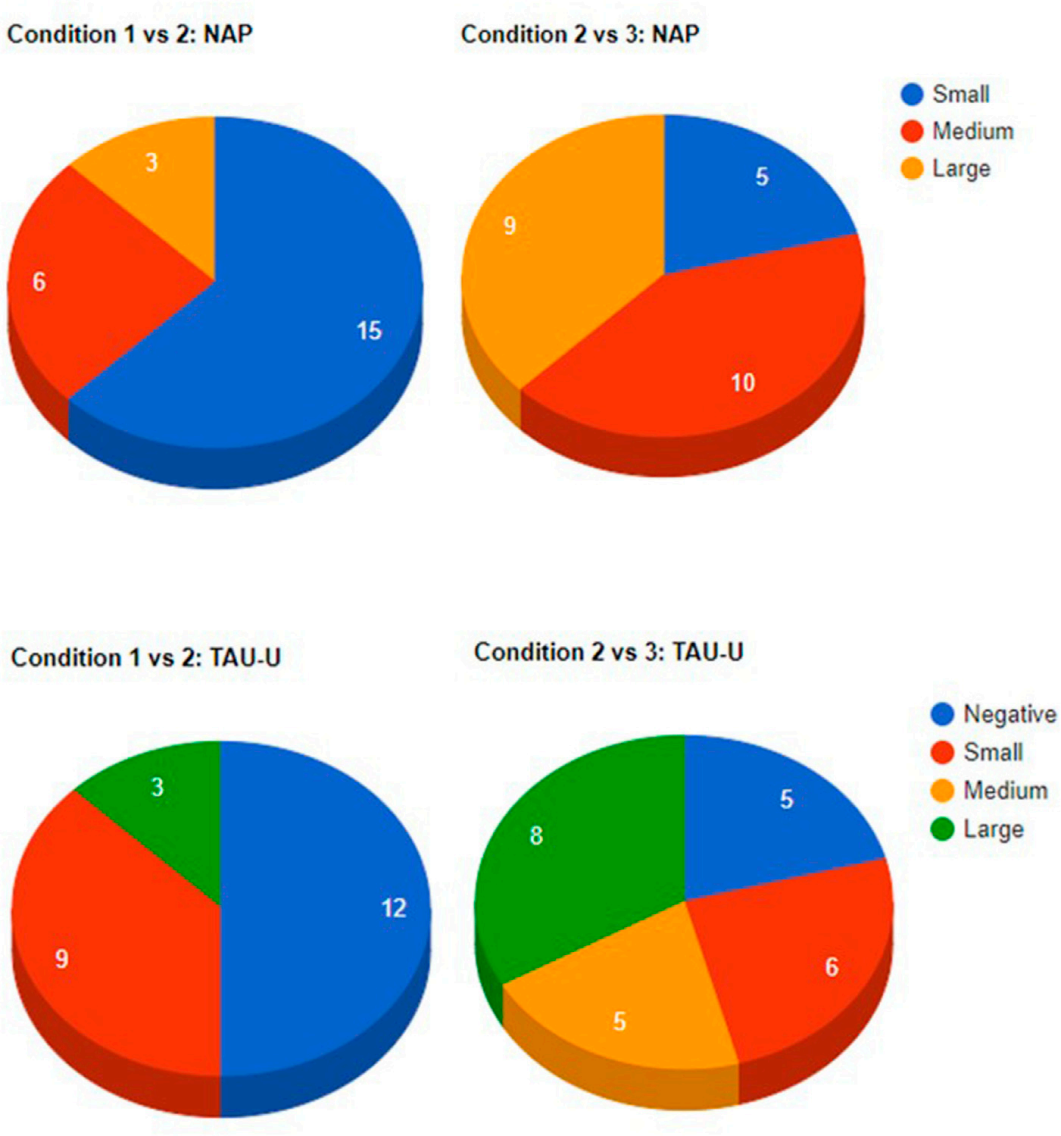
The top pie charts show the number of participants in each NAP effect size category when comparing Conditions 1 (no intervention) with Condition 2 (standard teaching) and Condition 2 with Condition 3 (robot intervention). The bottom pie charts show number of participants in each effect size category for TAU-U. NAP scores can range from 0 to 1, where values less than 0.66 are considered no or small effect, values between 0.66 and 0.92 a medium effect, and values of 0.93 and above a large effect. The same cut-off values were applied to TAU-U. Note that TAU-U values could also be negative, indicating that a condition change resulted in lower latencies as opposed to higher ones.

A summary of the effect size statistics is shown in Table 3. In comparisons of Conditions 1 and 2, means and medians were very similar. When comparing the latencies of Condition 2 with those of Condition 3, the median was noticeably higher than the mean. Overall, the values were higher for the second comparison than for the first. This difference was not so pronounced for NAP, but much clearer for TAU_novlap_ and TAU-U. Figure 1 provides an overview of the number of times these values met particular effect-size categories. For NAP, “small” indicated a value of less than 0.65, “medium” a value above 0.65 and below 0.93, and “large” a value of 0.93 and above. For TAU-U, values could also be negative. As TAU-U has the advantage over TAU_novlap_ that it adjusts for baseline trend, only TAU-U is shown here. For the comparison of Condition 1 with 2, therefore, TAU-U can adjust for the fact that there was a significant positive trend in Condition 1. The number of participants in medium and large NAP effect size categories was substantially higher when comparing Condition 2 with 3 than for Condition 1 *versus* 2 (Figure 1). For TAU-U, a similar pattern emerged. Transitioning from Condition 1 to 2 resulted in only three instances of a large effect size and none for medium, while transitioning from Condition 2 to 3 resulted in five medium effect sizes and eight large effect sizes.

**TABLE 3:** Effect size values (NAP, TAU_novlap_, and TAU-U) by participants for the transition from Condition 1 (no intervention) to Condition 2 (standard teaching) and the transition from Condition 2 to Condition 3 (robot intervention).

		Condition 1 vs. 2			Condition 2 vs. 3	
	NAP	TAU_novlap_	TAU-U	NAP	TAU_novlap_	TAU-U
S1	0.17	−0.67	−0.33	1.00	1.00	1.00
S2	0.67	0.33	0.17	0.89	0.78	0.67
S3	0.78	0.56	0.44	1.00	1.00	1.50
S4	0.67	0.33	0.17	1.00	1.00	1.50
S5	0.00	−1.00	−0.83	0.72	0.44	0.56
S6	1.00	1.00	1.00	0.44	−0.11	−0.22
S7	1.00	1.00	1.11	0.22	−0.56	−0.44
S8	0.00	−1.00	−1.25	1.00	1.00	1.00
S9	1.00	1.00	1.00	0.00	−1.00	−0.83
S10	0.00	−1.00	−0.83	1.00	1.00	1.17
S11	0.39	−0.22	−0.56	0.72	0.44	0.56
S12	0.50	0.00	−0.11	0.67	0.33	0.22
S13	0.22	−0.56	−0.89	1.00	1.00	0.89
S14	0.39	−0.22	−0.56	0.78	0.56	0.22
S15	0.17	−0.67	−0.78	0.89	0.78	0.89
S16	0.56	0.11	0.00	0.11	−0.78	−1.11
S17	0.67	0.33	0.00	0.94	0.89	1.00
S18	0.50	0.00	0.33	0.89	0.78	0.44
S19	0.33	−0.33	−0.22	1.00	1.00	1.11
S20	0.56	0.11	0.00	0.89	0.78	0.44
S21	0.67	0.33	0.00	0.22	−0.56	−0.22
S22	0.44	−0.11	−0.44	1.00	1.00	1.11
S23	0.11	−0.78	−0.89	0.78	0.56	0.67
S24	0.78	0.56	0.22	0.89	0.78	0.67
Mean	0.48	−0.04	−0.14	0.75	0.50	0.53
Median	0.50	0.00	−0.06	0.89	0.78	0.67

## Discussion

The present study explored the utility of robots as distractors in a delay-of-gratification task for preschoolers. In this single-case design study, 24 preschoolers completed three experimental conditions that differed in terms of availability of activities during the designated waiting period. During the first condition, where no activities were provided but the number of teachers present during the delay increased across sessions, children’s waiting times continuously increased. Waiting times did not increase further during the second condition when teachers offered activities such as reading, singing, or playing with toys. However, in the final condition, when children were able to observe and interact with robots, waiting times increased significantly. Whereas for most children the effect size for the transition from the first to the second condition indicated no intervention effect, slightly more than half of the participants exhibited a medium or large effect size during the robot intervention. These data indicate that exposure to robots appears beneficial as a distractor in a DG task for preschoolers.

Recent studies have increasingly applied robots in educational situations (Pandey and Gelin, 2017), including the teaching of self-regulation skills (Holtz and Lehman, 1995; Carlson, 2005; Lillard et al., 2015; Holeva et al., 2021). An important aspect of DG, however, is the ability to develop strategies to counter the effects of any frustration or preference reversal that may be occurring while actively choosing to wait for a preferred but delayed reward. Both self-distraction (Anderson, 1978) as well as distraction arranged by others (Putnam et al., 2002) have been shown to increase the likelihood of children exhibiting DG. Our pilot study has demonstrated that robots also appear to be an effective distractor.

The purpose of the present study was not to illustrate the superiority of robotic application as an intervention tool to teach DG, but rather to demonstrate its feasibility. Comparisons with traditional teaching methods such as those offered in Condition 2 were thus not the primary focus. More systematic comparisons between intervention approaches would have required counter-balancing of intervention order or a multiple-baseline design (Barlow et al., 2009), which was not feasible in the present preschool context. Instead, the goal was to explore to what extent the use of robots may provide a short-term beneficial effect in a -DG procedure in young children. The results demonstrated that it did, and in this case, the robot intervention appeared to provide additional benefits compared to Condition 2 (where latencies appeared to have reached stability). As educational interventions using robots continue to be developed further, DG may become a standard part of the educational delivery portfolio. Although DG has been suggested for robotic intervention previously (Ferrari et al., 2009), the feasibility of this approach had not yet been explored prior to the present study. As the functionality of robots continues to improve, future interventions will be able to assign an increasingly active role of robots in such procedures. We suggest that eventual robotic delivery of DGprograms will likely result in procedural consistency, accuracy of measurement, and cost saving in terms of staff time.

Although no measures of robot acceptance had been collected, informal feedback from teachers and parents indicated that the children generally enjoyed the interaction with the robots. The fact that waiting times in Condition 3 (robot intervention) increased across sessions indicates that the novelty of the robots is unlikely to have been the sole contributor to the comparatively high number of medium and large effect sizes, although the increasing levels of possible child-robot interactions across session may have played a role. Given logistical constraints and the need to restrict the length of the study at the school setting, we were unable to arrange a larger number of sessions in each condition and explore at what stage latency scores in the robot intervention condition were reaching a stable level. A further limitation is the lack of follow-up to explore to what extent the intervention effects were long lasting; that is, if children would continue to delay gratification even in the subsequent absence of the robots.

Additionally, our delay time was only a third of that in the original Mischel studies (e.g., Mischel et al., 1972). Although the majority of our participants did not reach the maximum wait times, some participants did, which raises the possibility that the effects we demonstrated may have been more pronounced at even longer delays. Particularly for children from families of higher socioeconomic status, longer wait times are commonly reported (Watts et al., 2018; Tunney, 2022). The children in our study may be considered as coming from predominantly medium to higher SES backgrounds within the society of Southern India. However, little is known about how DG may be different in children in India as compared to children in other countries and whether similar interaction effects with SES can be found there. Within the scant literature related to DG with participants in India are studies with adult participants on academic DG (Chakraborty and Chechi, 2019) or cognitive experiments exploring to what extent the so-called ego-depletion effect of self-control found in Western samples may generalize to India (Savani and Job, 2017). In the latter study, it was found that engagement in attentional control tasks did not have the same depleting effect in Indian participants’ ability to perform well in immediately-following cognitive tasks. Savani and Job (2017) argued that this effect of *reverse ego-depletion* indicates that the exertion of willpower may have an energizing effect in cultures such as India where religious and philosophical traditions tend to emphasize the application of mental self-control to everyday life. To what extent this also applies to the values embedded within Indian educational contexts for young children needs to be explored in future research. Other factors to be explored include to what extent DG in preschoolers in India tend to be specific to types of reward used: Yanaoka et al. (2022), for example, found that children in Japan were more likely to show DG for food items than children in the United States, who in turn had longer waiting times for gifts than Japanese children. Eventual success in applying robotic technology in the teaching of DG ultimately depends on the ability to generate higher efficiency than what current approaches can offer. Particularly for DG, where differences regarding SES can already be found (Watts et al., 2018; Tunney, 2022), unequal deployment across economic lines would only exacerbate SES-related disparity in self-control abilities. In the application of robotics in health fields, affordability has been recognized as a crucial factor to ensure equitable access to health services, thus influencing the direction of technological development (Chamzas et al., 2022; Matsumoto et al., 2022). The importance of equity has also been recognized in calls for policy around the use of robotic technology in public spaces (Mintrom et al., 2022) as well as education (Kupferman, 2022).

Further limitations of the present study need to be noted. Given time constraints, Condition 1 was not run until stability had been reached, and possibly the addition of the teachers across sessions affected waiting time (e.g., children looking for approbation for waiting). In Condition 3, the intensity of the intervention (extent of possible interaction with robots) increased across sessions, which, as we note above, may have been partly the reason for the positive trend in that condition. The trend during Condition 1 did not continue into Condition 2, and effect size calculations were able to adjust for trend using the measure TAU-U. For Condition 3, effect sizes may have been larger if the condition had contained more sessions. Future studies thus need to replicate the study with longer conditions. Of course, increasing the number of sessions will eventually create experimental fatigue or a ceiling effect if sufficient DGskills have already been acquired by the participants. A further limitation of the present study was that the rewards used varied to a very high extent, which may have resulted in variability of waiting time results. Previous studies have limited the choice of rewards to two items (Mischel et al., 1972). However, our highly individualized choice may have increased the likelihood that the value of the reward was indeed very high for the participants. Lastly, our sample included children in the age range 4 or 5 years, as opposed to 3 to 6 in the study by Mischel et al. (1972), for example,. Our results are therefore not directly comparable to those reported in other studies, and future studies with robot interventions will need to explore the suitability of the intervention approach in other age groups as well as other measures of self-regulation and other outcome variables.

To summarize, the present study has demonstrated the possible utility of robots as a distraction when studying DG in preschoolers. We emphasize that our goal was merely to investigate the feasibility of a robotic intervention in principle, as a starting point for future studies. We do note, however, that compared to no intervention or standard teaching, exposure to robots during the self-imposed waiting period increased the likelihood of participants waiting for the delayed, more preferred reward as opposed to consuming a smaller, immediate reward. The medium and large effect sizes for the robot intervention indicate that this approach has merit in the development of future, more automized robotic interventions.

## Data Availability

The original contributions presented in the study are included in the article/supplementary material, further inquiries can be directed to the corresponding author.
